# Chemsex and Sexual Well-Being in Young Polish Men

**DOI:** 10.3390/ijerph20126163

**Published:** 2023-06-17

**Authors:** Rafał Gerymski, Wiktoria Magoń

**Affiliations:** 1 Department of Health Psychology and Quality of Life, Institute of Psychology, Opole University, 45-040 Opole, Poland; 2 Institute of Psychology, Opole University, 45-040 Opole, Poland

**Keywords:** chemsex, sexual well-being, sexual satisfaction, life satisfaction, perceived stress

## Abstract

Chemsex refers to the use of psychoactive drugs for sexual purposes—before or during sex. This phenomenon mainly affects men, in particular those belonging to the LGBTQIA+ (lesbian, gay, bisexual, transgender, intersex, queer/questioning, asexual, and more diverse individuals) community. From the perspective of the transactional theory of stress, chemsex can be considered a strategy for coping with stress, which is why it is also extremely important to verify its role in functioning outside the sexual sphere. For this reason, this study verified the relationship between the use of chemsex, perceived stress, sexual well-being, and life satisfaction in young Polish men. The study involved 175 men (67 people using chemsex and 108 people in the control group) between 18 and 33 years of age. The Perceived Stress Scale, the Short Scale of Sexual Well-being, the Satisfaction with Life Scale, and the authors’ questionnaire about the use of chemsex were used. It was observed that individuals using chemsex showed a significantly lower level of sexual well-being and satisfaction with life (moderate effects) and a higher level of perceived stress (strong effect) when compared to the control group not using psychoactive substances. Additionally, a positive and moderate relationship was observed between the number of psychoactive substances used and perceived stress in the group of individuals using chemsex. Moreover, the number of substances used and the level of perceived stress were negatively and moderately related to the level of well-being in these individuals. It was also shown that perceived stress was a significant predictor of the number of psychoactive substances used before and during sex and that perceived stress and the number of psychoactive substances used were significant and negative predictors of life satisfaction and sexual well-being, explaining a large portion of their variance.

## 1. Introduction

Chemsex refers to the use of psychoactive drugs for sexual purposes—before or during sex. These substances can be used to inhibit control, feel more sexually aroused, or have more and longer sexual encounters. In an intoxicated state, the thoughts, emotions, and sensations that prevent free sexual behavior can be abolished [1,2]. The effect of psychoactive substances is mainly based on neurotransmitter systems. They can be responsible for relaxation, well-being, euphoria, and the elimination of unpleasant effects. Nevertheless, the multifunctionality of a substance is not necessarily a positive property because if it is possible to affect various neurotransmitter systems simultaneously with one stimulant, it also means that opposing mechanisms can often be activated. The most important systems include serotonergic, dopaminergic, and noradrenergic systems, and it is in them that the reuptake of named neurotransmitters is blocked, e.g., after taking cocaine. The multifunctionality of neurotransmitters has been demonstrated by dopamine, the transmission of which is carried out through the projections of the following pathways: mesolimbic, mesocortical, nigrostriatal, and nodules-funnel. Due to such a wide transmission, dopamine can affect the ventral tegmental nucleus (belonging to the reward system) but also the substantia nigra. This reward system is involved in the regulation of mood and motivation, while the substantia nigra is fundamentally involved in human motor coordination [3]. Therefore, due to the multitude of functions performed by neurotransmitter systems, it is difficult to determine what the effect would be of stimulating them with high strength at the same time. It is important to emphasize that long-term stimulations and the application of certain types of stimuli can lead to neuroplastic changes that often are responsible for addiction [4].

Psychoactive substances are most often taken before or during sex to maintain or increase sexual sensations or to relieve the physical and mental discomfort associated with it. An important reason is also gaining self-confidence, which can improve sexual performance [5]. The most commonly used substances include marijuana, cocaine, mephedrone, crystallized methamphetamine, gamma-hydroxybutyrate, gamma-butyrolactone, 1,4-butanediol, alkyl nitrites (poppers), and alcohol, which are sometimes used in combination with non-psychoactive drugs that are used to treat erectile dysfunctions, such as sildenafil, tadalafil or vardenafil [1,2,5,6,7]. It is important to point out that the use of various substances, in the long run, can bring negative health effects. The excessive activation of neurotransmitter secretion, blocking reuptake, and excessive biosynthesis of neurotransmitters, apart from causing desired effects in the form of euphoria, relaxation, and hallucinations, can also cause the dysregulation of the functioning of mitochondria, the degeneration of neurons and their processes, and even apoptosis [3].

Stuart [5] points out that the chemsex phenomenon is particularly popular among homosexual and bisexual men. The greater popularity of chemsex among men may result from their more frequent use of psychoactive substances, not only for sexual purposes. Studies on the Polish population have shown that men more often than women reach for psychoactive substances [8]. The more frequent use of psychoactive substances by men is also consistent with previous research on stress-coping strategies. Women more often choose strategies that are focused on expressing emotions, venting them on others, and seeking social support. Men, in turn, more often cope with stress by reaching for alcohol and drugs [9,10]. In the scientific literature, chemsex is often identified with a group of men who sleep with other men (men who have had sex with men; MSM). A meta-analysis of 23 articles on the Asian population in 2023 indicated the particular popularity of the chemsex phenomenon in the MSM group [11]. Nevertheless, a review of the epidemiological data also shows that this phenomenon is not limited to the LGBTQIA+ (lesbian, gay, bisexual, transgender, intersex, queer/questioning, asexual, and more diverse individuals) community. One of the largest chemsex studies to date was conducted in 2019 [12] on a population of over 22,200 people. In this study, above 82% of men and 77% of women identified themselves as heterosexual. Chemsex behavior was observed in each group of individuals, and the results indicated significant differences in the preferences of the psychoactive substances used for chemsex. Chemsex was still most common in the MSM group but was not limited only to this group of individuals. For example, women, regardless of their sexual orientation, more often reported having sex while under the influence of alcohol [12]. However, research data are not consistent with Stuart’s [5] statement that chemsex is particularly popular among non-heterosexual men. Studies conducted on a population of over 2000 MSM in Scotland, Wales, Northern Ireland, and the Republic of Ireland showed that only 6% of the studied MSM individuals engaged in substance-linked sex [13].

The current state of research on chemsex does not allow for the formulation of coherent conclusions. Data on the relationship between chemsex and well-being are conflicting and unclear. The research by Rolt [14] showed that 64% of people using chemsex showed that it had a positive impact on their quality of life and sexual satisfaction. The group that reported the negative effects of chemsex on their lives were men with high levels of stress and a low sense of control over substance use. On the other hand, in a study by Hibbert et al. [7], 83% of MSM who used chemsex enjoyed their sex life and felt in control of it. Another 89% of MSM who used alcohol reported the same feelings, although importantly, both groups were characterized by lower satisfaction with life. What is more, studies on the Czech population showed that MSM using chemsex had significantly higher sexual satisfaction than MSM not using any substances [15]. These studies are inconsistent regarding how chemsex affects the well-being of people who engage in it. For this reason, attempts to determine the direction of interactions should be sought in theory. One of the main paradigms of research on coping with stress is the transactional stress model of Lazarus and Folkman [16]. According to the transactional theory of stress, the events in a person’s life are evaluated and can be assessed as threatening or non-threatening. In a situation that brings a sense of threat, strategies for coping with stress (in short: coping) are activated and act as a mediator between the stressor and the effects of coping with stress (e.g., health or well-being). In the case of constructive coping strategies, the results of stress transactions are usually positive (e.g., an increase in well-being). In the case of using destructive coping strategies (such as the use of psychoactive substances) or ineffective coping, an individual’s well-being most often deteriorates [16]. Based on the presented theoretical framework, it can also be concluded that the use of chemsex acts as a strategy for coping with stress, which can bring negative effects. The role of chemsex in the transactional stress theory paradigm has not yet been explored, but similar research is available on the use of psychoactive substances outside the sexual sphere. Wilchek-Aviad and Oren [17] showed that substance use was significantly associated with LGBT minority stress. In contrast, studies by Young and colleagues [18] found that sex workers were more likely to use psychoactive substances to increase self-confidence, control, and intimacy with others and to reduce feelings of guilt and sexual distress in comparison to women who were not sex workers. These results suggest that chemsex could be operationalized as a destructive coping strategy. 

Long-term substance use can lead to addiction. The reward system, which is essential for feelings of pleasure, under the constant use of psychoactive substances can become dysfunctional [4]. Koralewska-Samko, Sadowska, and Spryszyńska [19] wrote about the destructive impact of substance use, which showed that taking psychoactive substances was a form of coping with stress and could be associated with an increase in aggression in this group of adolescents. Additionally, among young adults, those who smoked marijuana and drank alcohol as part of a coping strategy were less likely to cope with frustration and their own emotions. What is also important is that these people tended to initiate intentional suffering when they experienced failures and failures more often [20]. 

Because functioning in the sexual sphere is an important predictor of life satisfaction [21], in this study, it was decided to verify the role of chemsex as a mediator in the relationship between perceived stress and life satisfaction and sexual well-being in young Polish men. Based on the presented research results and the transactional model of stress, it was assumed that: (1) perceived stress is negatively related to life satisfaction and sexual well-being, and (2) chemsex acts as a mediator in the relationship between perceived stress, life satisfaction, and sexual well-being.

## 2. Materials and Methods

The presented study is part of a larger project focusing on sexual well-being in the Polish community, conducted by the first author (R.G.). It received a positive recommendation from the Research Ethics Committee at the affiliation of the first author (application number: KEBN 1/2021). Each of the study participants was informed about the full anonymity of the obtained data and the possibility of stopping participation in the study at any time without giving a reason. The presented chemsex study was carried out in two phases.

### 2.1. Study 1 and Its Participants

The first study was focused on the recruitment of chemsex users. Access to these respondents was obtained via social media. The study involved 67 men between 18 and 31 years of age. Unfortunately, it was not possible to recruit a number of women that was sufficient to carry out statistical analyses. For this reason, only the results of men were included in the presented study. The exact characteristics of the study sample are presented in Table 1. The inclusion criteria were as follows: (1) aged over 18 years old; (2) no declared hospitalization in a psychiatric ward in the last 30 days; (3) the possibility of expressing informed consent to participate in the study; (4) the use of chemsex in the last month prior to the study.

### 2.2. Study 2 and Its Participants

In the second study, a control group with similar characteristics to the sample obtained in the first study was recruited. In total, 108 men aged between 18 and 33 years of age took part in it. The exact characteristics of the study sample are presented in Table 1. The study included the results of people who met the following requirements: (1) aged over 18; (2) no declared hospitalization in a psychiatric ward in the last 30 days; (3) the possibility of expressing informed consent to participate in the study; (4) not using psychoactive substances (even alcohol) and not practicing chemsex throughout one’s whole life.

### 2.3. Measures

The study measured four variables. Three validated questionnaires and one author’s questionnaire were used. Stress levels were measured with the Perceived Stress Scale (PSS-10) [22]. This consisted of 10 questions on a 5-point scale, where: 0—“never”; 4—“very often”. A higher PSS-10 score indicated higher subjectively perceived stress. In the presented study, the PSS-10 questionnaire obtained good reliability (Cronbach’s alpha = 0.89).

Sexual well-being was measured using the Short Sexual Well-Being Scale (SSWBS) [23]. It consisted of 5 questions on a 7-point scale, where: 0—“I strongly disagree”; 7—“I strongly agree”. A higher SSWBS score indicated a higher level of subjective sexual well-being. In the presented study, the SSWBS questionnaire obtained good reliability (Cronbach’s alpha = 0.92).

The Satisfaction with Life Scale (SWLS) [24] was also used. It consisted of 5 questions on a 7-point scale, where: 1—“I strongly disagree”; 7—“I strongly agree”. A higher SWLS score indicated a higher subjective assessment of life satisfaction. In the presented study, SWLS obtained good reliability (Cronbach’s alpha = 0.88).

Chemsex data were measured using the authors’ questionnaire. It consisted of an open-field question about the range of psychoactive substances used by the study participants before or during sex. The respondents were not provided with a list of proposed substances—individuals completing the questionnaire had to independently write down the substances they had used in the last 30 days for sexual purposes. The exact data on this subject are presented in Table 1 in the results section.

### 2.4. Data Analysis Procedure

Data from both studies were collected via the Internet. All data were stored only in a digitized form on the personal computer of the first author (R.G.) and his external drives for data protection. No one other than the authors of this manuscript had access to the collected data. All results were analyzed using IBM SPSS Statistics 28 (Predictive Solutions Sp. z o.o., Cracow, Poland). Differences between the studied groups were verified with chi-squared and t-test analyses. The relationships between the studied variables were measured with Pearson’s r correlation and mediation analysis performed with the PROCESS macro [25] for SPSS software. A level of α = 0.05 was adopted as the threshold value for statistical significance in all the analyses.

### 2.5. Sensitivity Power Analysis

Many commonly used measures of statistical significance, such as the *p*-value, are strongly dependent on the size of the surveyed sample of people [26]. When discussing the results of this study, effect size measures were used because they were independent of the size of the tested sample of the respondents [27,28,29]. In connection with the above, a sensitivity power analysis was carried out. The presented sample of 67 people using chemsex allowed for reliable conclusions about effects greater than ρ = 0.32 (in the case of correlations) and ƒ^2^ = 0.12 (in the case of regression analyses), both at the level of α = 0.05 and the power of 1-β = 0.80. This means that based on the results of the presented group, one should not conclude about low effects that are smaller than the indicated value of the presented coefficients. In the case of the group comparison between people using chemsex (*n* = 67) and the control group (*n* = 108), the analysis showed that the results of Cohen’s d coefficient exceeded the value of 0.44 (low effect), which should be considered reliable.

## 3. Results

### 3.1. Characteristics of the Tested Sample

Table 1 presents the characteristics of the samples from both studies. The group using chemsex included 65 cisgender men and 2 individuals who declared themselves transgender men. In the control group, 103 cisgender men and 5 transgender men were recruited. None of the respondents declared themselves as non-binary individuals, and the labels presented in Table 1 come directly from the data declared by the respondents. In both groups, the dominant majority were people living in cities, in an informal relationship, or who were single, most often working (1:1 ratio in the control group), and with higher than secondary education. Significant differences were noticed in the case of declared sexual orientation. The chi-squared analysis showed that in the group of people using chemsex, the percentage of homosexual men was significantly higher than in the control group, where heterosexual men were the dominant group (chi-squared = 53.71; *p* < 0.001; Cramer’s V = 0.55—strong effect). The most commonly used chemsex-related substances in the first study’s group were poppers, marijuana, alcohol, and psilocybin mushrooms (see Table 1).

### 3.2. Group Differences

To compare the level of perceived stress and well-being in the tested groups, a t-test for independent samples analysis was used. It was observed that individuals using chemsex showed a significantly lower level of sexual well-being and satisfaction with life (moderate effects) and a higher level of perceived stress (strong effect) when compared to the control group not using psychoactive substances. The exact data are presented in Table 2. Due to the size of the presented subgroups in the socio-demographic variables (see Table 1), they were not used in statistical analyses.

### 3.3. Relationships between Tested Variables

In the next step, Pearson’s r correlation analysis was performed to verify the relationships between the tested variables. A positive and moderate relationship was observed between the number of psychoactive substances used and perceived stress in the group of individuals using chemsex. Moreover, the number of substances used and the level of perceived stress were negatively and moderately related to the level of well-being in these individuals. In the case of the control group, a negative and moderately strong relationship was also observed between perceived stress, life satisfaction, and sexual well-being. The exact data are presented in Table 3.

To verify the mediating role of chemsex, an analysis using the PROCESS macro was used [25]. MODEL 4 was used in this analysis using the bootstrapping method with a declared number of 5000 samples [30]. The analysis showed that perceived stress was a positive predictor of the number of psychoactive substances used before and during sex—the higher the stress, the greater the amount of declared chemsex-related substances. Moreover, the perceived stress and the number of psychoactive substances used before and during sex were significant predictors of life satisfaction and sexual well-being. Along with an increase in the results of perceived stress and the greater declared number of psychoactive substances, there was a decrease in the results of satisfaction with life and sexual well-being. Unfortunately, the verification of the mediating role of chemsex turned out to not be possible. A Monte Carlo power analysis for the indirect effects proposed by Schoemann et al. [31] showed that the tested sample was not sufficient for the tested indirect effects to be reliably verified (1-β = 0.45/0.47, respectively). The calculated ƒ2 measures exceeded the cut-off criteria of 0.12 for the regression analyses calculated in the sensitivity power analysis. This meant that only the tested direct effects could be further discussed. The exact data are presented in Table 4.

## 4. Discussion

This study aimed to verify the relationship between chemsex, perceived stress, life satisfaction, and sexual well-being. The results showed that men using chemsex reported higher levels of perceived stress and lower levels of life satisfaction and sexual well-being. It was also shown that (1) perceived stress was a significant predictor of the number of substances used before and during sex, and (2) the number of psychoactive substances used and perceived stress were negative predictors of life satisfaction and sexual well-being in individuals using chemsex.

Lower well-being and higher levels of stress in the group using chemsex might not only be related to the use of these substances but also to other significant variables. In the presented study, chemsex was mostly used by people from the LGBTQIA+ community. According to the European Union Agency for Fundamental Rights (FRA), Poland is a country where people very often suffer from discrimination or harassment regarding their sexual orientation or belonging to the LGBTQIA+ community [32]. The FRA report shows that homosexual men accounted for the largest percentage of respondents (68%) in terms of experiencing violence in the last 12 months due to belonging to the LGBTQIA+ community. What is more, Polish research also showed that transgender men and MSM had a lower quality of life than heterosexual cisgender men [33]. Therefore, the relationship between chemsex, stress, and well-being requires more thorough verification while controlling for such variables as perceived heterosexism or minority stress.

Nevertheless, chemsex could likely have been used in the study sample as a strategy for coping with stress. A German study showed that men practicing chemsex experienced depression, anxiety, and somatization states significantly more often than men who did not use psychoactive substances for sexual purposes. However, these data should be treated with caution, as the study groups did not differ significantly in terms of the occurrence of clinical symptoms for the listed disorders [34]. It is difficult to determine the direction of existing correlations because stressors could lead to the practice of chemsex when eliminating the unpleasant effects of a stressful situation; however, there is also the possibility that chemsex itself causes negative side effects in various forms, such as depression, anxiety, and unpleasant somatic changes. This is extremely important because psychoactive substances are not neutral for the body, and their intake, apart from the pleasant effects of stimulating the reward system, brings several serious side effects [4]. Nevertheless, regarding the transactional model of stress [16], this group of individuals may likely have used chemsex either as a form of coping with stress or in connection with ineffective coping and affective problems. Unfortunately, neither the presented study nor other available data allow us to verify this supposition; therefore, as the next steps, the reasons behind the use of psychoactive substances for sexual purposes in the Polish population should be thoroughly analyzed.

Taking substances for sexual purposes may be a form of coping with stress in the minority group, bringing negative effects. Polish research has shown that traditional coping strategies did not buffer the relationship between perceived stress and life satisfaction in transgender individuals and also transgender men [35]. It is probable that in the minority men sample, chemsex is a form of coping, negatively shaping the well-being of these people. Psychoactive substances accompanying sexual contact are intended to maximize pleasure. Nevertheless, this means that there are pre-existing factors that reduce sexual satisfaction. In addition, combining sexual pleasure with the use of substances may facilitate drowning out the factors that reduce sexual performance. Taking psychoactive substances as a form of coping with stress is not an effective method because the effect of taking it in the form of positive effect is temporary. It is also a dangerous method for coping, as neuroplastic changes can lead to addiction. The mechanisms of individual substances are not fully understood, and the effect of taking such a substance is extremely strong and unpredictable in its consequences [4]. Taking psychoactive substances is a destructive and maladaptive strategy for coping with stress in various age groups [19,20]. It allows the suppression of factors that weaken sexual performance and result from various types of stressors. However, the pleasure of substance-enhanced sex can be temporary because the stressor underlying the inability to achieve sexual satisfaction while sober is still present. In addition, long-term substance use poses a significant risk of addiction and the weakening of executive functions, which can be associated with unpleasant affective changes [4]. Therefore, future studies on chemsex should keep verifying its role from a coping point of view.

The presented results show the effects of a pilot study. It provides data that can encourage nationwide research to verify the role of chemsex in sexual and non-sexual functioning. It is the first to verify the role of chemsex in the perspective of the transactional stress theory. However, despite its innovative character, this study is not free from limitations. Firstly, the group of women was not included in the presented study. Secondly, the presented group of people was not very large, which is a barrier in comparison to the sociodemographic variables and prevented the verification of indirect effects in the mediation analysis. Moreover, the recruited sample of people using chemsex did not meet the criteria of representativeness; therefore, the presented results cannot be extrapolated to the entire population of Poland. The presented project also failed to measure important variables, such as a diagnosis of addiction to psychoactive substances, the frequency of using chemsex, motives for reaching chemsex, or the level of perceived heterosexism and minority stress in a sample of individuals from the LGBTQIA+ community. Lastly, the effects of chemsex on one’s health and well-being are multiple and often unpredictable [4]. Its impact on individuals’ functioning should be studied, taking into account the effect and impact of each chemsex-related substance separately. In the presented results, this was impossible due to the low popularity of some chemsex-related substances, and only a number of used psychoactive substances were analyzed. Therefore, future studies on the chemsex phenomenon should strive to eliminate this limitation and analyze the effects of individual substances separately. Despite the aforementioned numerous limitations, the study is innovative and is an important contribution to further research on the role of chemsex in the functioning of Polish individuals.

## 5. Conclusions

The presented results show that chemsex can be significantly related to levels of perceived stress, sexual well-being, and life satisfaction in Polish men. What is more, it was underlined that individuals using chemsex had a significantly lower level of sexual well-being and satisfaction with life and a higher level of perceived stress when compared to a control group not using psychoactive substances. These results indicate that people involved in chemsex may function worse in the sphere of mental and sexual health in relation to people not using psychoactive substances before and during sex. This indicates an important practical implication for those working with individuals involved in chemsex, and MSM in particular. Clinical practitioners and supporting allies should particularly care for the well-being of individuals engaged in chemsex because, as shown in this study, it might be associated with more limited functioning in the mental and sexual spheres.

Research on chemsex in the paradigm of the transactional model of stress by Lazarus and Folkman [16] should be repeated, taking into account a larger sample of respondents (including women) and taking into account such variables as minority stress and heterosexism in the case of individuals from the LGBQIA+ community. This is extremely important because if the study participants use chemsex as a destructive form of coping, preventive steps should be taken to educate them on the constructive forms of dealing with stress. The use of chemsex likely brings short-term improvements in well-being, but in the long term, it is not a constructive coping strategy. Due to the small amount of scientific evidence and contradictory conclusions from available studies, research on chemsex should be continued, not only in Poland.

## Figures and Tables

**Table 1:** Characteristics of the tested sample.

		Chemsex Group		Control Group	
		*M*	*SD*	*M*	*SD*
Age		27.46	8.83	24.33	7.33
		*n*	% (within the Group)	*n*	% (within the Group)
Gender identity					
	Men	65	97.01%	103	95.37%
	Transgender men	2	2.99%	5	4.63%
Place of residence					
	Town/city	55	82.09%	80	74.07%
	Village	12	17.91%	28	25.93%
Marital status					
	Informal relationship	33	49.25%	49	45.37%
	Single	22	32.84%	52	48.15%
	Married	11	16.42%	7	6.48%
	No data	1	1.49%	0	0.00%
Employment status					
	Employed	45	67.16%	54	50.00%
	Unemployed	22	32.84%	54	50.00%
Education					
	Secondary	33	49.25%	61	56.48%
	Higher	25	37.31%	30	27.78%
	Basic	9	13.43%	12	11.11%
	Vocational	0	0.00%	5	4.63%
Sexual orientation					
	Homosexual individual	43	64.18%	13	12.04%
	Heterosexual individual	17	25.37%	77	71.30%
	Bisexual individual	5	7.46%	15	13.89%
	Pansexual individual	2	2.99%	2	1.85%
	No data	0	0.00%	1	0.93%
Chemsex-related substance use in the past 30 days					
	Poppers	37	55.22%	-	-
	Alcohol	30	44.78%	-	-
	Marihuana	30	44.78%	-	-
	Psilocybin mushrooms	21	31.34%	-	-
	Opioids	16	23.88%	-	-
	MDMA	10	14.93%	-	-
	Amphetamine	8	11.94%	-	-
	LSD	6	8.96%	-	-
	Designer drugs	3	4.48%	-	-
	GHB (“rape pills”)	3	4.48%	-	-

Note: Due to the size of the presented subgroups, socio-demographic variables were not used in statistical analyses.

**Table 2:** Comparison of the studied groups—*t*-test for independent samples.

	Chemsex Group		Control Group		*t* (173)	*p*	*LLCI*	*ULCI*	*d*
	*M*	*SD*	*M*	*SD*					
Perceived stress	21.55	7.68	14.85	8.18	5.47	<0.001	4.279	9.112	0.84
Sexual well-being	17.10	8.16	21.50	8.15	−3.47	0.001	−6.898	−1.893	0.54
Life satisfaction	16.34	7.32	20.00	5.97	−3.61	<0.001	−5.657	−1.656	0.55

**Table 3:** Relationships between the studied variables—Pearson’s r correlation.

Chemsex Group	*M*	*SD*	1.	2.	3.
Chemsex	2.09	0.93	-		
2. Perceived stress	14.85	8.18	0.36 **	-	
3. Sexual well-being	17.10	8.16	−0.39 **	−0.55 ***	-
4. Life satisfaction	16.34	7.32	−0.38 **	−0.50 ***	0.40 **
Control group	*M*	*SD*	1.	2.	3.
-	-	-	-		
2. Perceived stress	21.55	7.68	-	-	
3. Sexual well-being	21.50	8.15	-	−0.35 ***	-
4. Life satisfaction	20.00	5.97	-	−0.62 ***	0.47 ***

Note: ** *p* < 0.01; *** *p* < 0.001; chemsex should be understood as the number of substances used before and during sex in the past 30 days.

**Table 4:** Verification of indirect effects—mediation analysis of the PROCESS macro (Model 4).

Perceived Stress → Chemsex → Life Satisfaction			*R^2^*	*ƒ* ^2^	*Beta*	*p*	*LLCI*	*ULCI*
Perceived stress	→	Chemsex	0.36	0.56	0.36	0.003	0.015	0.067
Chemsex	→	Life satisfaction	0.59	1.44	−0.23	0.048	−3.526	−0.011
Perceived stress (Chemsex)	→	Life satisfaction			−0.42	<0.001	−0.579	−0.178
The indirect effect of chemsex			-	-	−0.08	-	−0.263	0.003
**Perceived stress** **→ Chemsex** **→ Sexual well-being**			*R^2^*	*ƒ* ^2^	*Beta*	*p*	*LLCI*	*ULCI*
Perceived stress	→	Chemsex	0.36	0.56	0.36	0.003	0.015	0.067
Chemsex	→	Sexual well-being	0.59	1.44	−0.22	0.046	−3.815	−0.032
Perceived stress (Chemsex)	→	Sexual well-being			−0.47	<0.001	−0.686	−0.255
The indirect effect of chemsex			-	-	−0.08	-	−0.247	0.003

Note: chemsex should be understood as the number of substances used before and during sex in the past 30 days.

## Data Availability

Data will be made available from the corresponding author upon reasonable request.
